# Effect of Different Crosslinkers on Denatured Dentin Collagen’s Biostability, MMP Inhibition and Mechanical Properties

**DOI:** 10.3390/polym15183683

**Published:** 2023-09-07

**Authors:** Saleha Nisar, Viviane Hass, Rong Wang, Mary P. Walker, Yong Wang

**Affiliations:** School of Dentistry, University of Missouri—Kansas City, 650 E 25th St., Kansas City, MO 64108, USA

**Keywords:** denatured dentin, heat denaturation, acid denaturation, collagen, crosslinking, flavonoids, collagen hybridizing peptide, FTIR, weight loss, hydroxyproline assay, matrix metalloproteinases

## Abstract

Objective: Sound, natural dentin collagen can be stabilized against enzymatic degradation through exogenous crosslinking treatment for durable bonding; however, the effect on denatured dentin (DD) collagen is unknown. Hence, the ability of different crosslinkers to enhance/restore the properties of DD collagen was assessed. Methods: Demineralized natural and DD collagen films (7 mm × 7 mm × 7 µm) and beams (0.8 mm × 0.8 mm × 7 mm) were prepared. DD collagen was experimentally produced by heat or acid exposure, which was then assessed by various techniques. All specimens were then treated with 1 wt% of chemical crosslinker 1-ethyl-3-(3-dimethylaminopropyl) carbodiimide/n-hydroxysuccinimide (EDC/NHS) and two structurally different flavonoids—theaflavins (TF) from black tea and type-A proanthocyanidins from cranberry juice (CR) for either 30 s or 1 h. The controls were untreated. Dentin films were assessed for chemical interaction and cross-linking effect by FTIR, biostability against exogenous collagenase by weight loss (WL) and hydroxyproline release (HYP), and endogenous matrix metalloproteinases (MMPs) activity by confocal laser microscopy. Dentin beams were evaluated for tensile properties. Data were analyzed using ANOVA and Tukey’s test (α = 0.05). Results: Compared with natural collagen, DD collagen showed pronounced structural changes, altered biostability and decreased mechanical properties, which were then improved to various degrees that were dependent on the crosslinkers used, with EDC/NHS being the least effective. Surprisingly, the well-known MMP inhibitor EDC/NHS showed negligible effect on or even increased MMP activity in DD collagen. As compared with control, cross-linking induced by TF and CR significantly increased collagen biostability (reduced WL and HYP release, *p* < 0.05), MMP inhibition (*p* < 0.001) and mechanical properties (*p* < 0.05), regardless of denaturation. Conclusions: DD collagen cannot or can only minimally be stabilized via EDC/NHS crosslinking; however, the challenging substrate of DD collagen can be enhanced or restored using the promising flavonoids TF and CR.

## 1. Introduction

Dentin collagen that forms the body of the tooth, is a structurally complex substrate which is challenging to restore. The organic matrix of dentin is composed of type I collagen which forms a 3-dimensional network stabilized through inter and intramolecular crosslinking [1,2]. However, this stable, resilient structure is susceptible to degradation due to a low pH caused by either caries or by the acid etching procedure that initiates a vicious cycle of endogenous enzymes activation leading to breakdown of the collagen molecules [3]. These enzymes, such as matrix metalloproteinases (MMPs) can bind to specific sites in the collagen molecules and cause unfolding of the triple helix structure [4]. This results in loss of crosslinks at inter and intramolecular levels causing structural alterations within the collagen molecules which further leads to compromised mechanical properties and collagen stability [5].

To counteract collagen degradation, induction of exogenous crosslinks has been proposed to provide stability to the collagen structure and stop the vicious cycle of degradation [6]. Naturally derived flavonoids such as proanthocyanidins (PAC) have been shown to effectively provide stabilization to dentin collagen exposed to enzymatic challenges [7,8]. Type-A PAC from cranberry juice (CR) can directly inhibit endogenous enzymes present in the dentin matrix and play a crucial role in reducing collagen degradation [9]. While one study has rendered CR’s performance weak [8], a more recent study showed that it could provide resistance to enzymatic degradation similar to the gold standard type-B PAC [7]. Another natural crosslinker, structurally different flavonoid theaflavin (TF) from black tea, is a comparatively smaller compound than PAC and has shown promising results when tested for dentin collagen biostability [10]. Both these crosslinkers were able to crosslink natural dentin collagen in a clinically feasible time-period of 30 s. Unlike CR or TF whose crosslinking mechanisms with collagen involve various multi-scale interactions including hydrogen, hydrophobic or chemical bonding; chemical crosslinker EDC/NHS is a zero length crosslinker that creates crosslinks in the dentin collagen without any molecules being part of the final bonds created [11]. Specifically, amide bonds, facilitated by EDC/NHS, are formed between carboxylic acids and amine groups locally available from side chains of collagen polypeptides. This crosslinker was able to produce an inhibition of endogenous enzymes as well as an improvement in the mechanical properties of dentin when used for longer treatment time [12,13]. A recent study further has shown high interfacial bond strengths between two materials (dentin and adhesive) and low activity of endogenous enzyme following storage of samples treated with EDC primer for 5 years [14]. The promising effects of these crosslinkers have been tested on sound natural dentin and information from clinical settings regarding their effects on denatured dentin (DD) collagen from dentin substrates is scarce. In particular, it is unknown whether the different crosslinking mechanisms of the above crosslinkers would affect their ability to stabilize structurally altered DD collagen.

DD collagen is frequently encountered in clinically relevant substrates. For example, dentin surfaces that have been prepared with cutting or abrading instruments are normally covered with a 0.5–2-µm-thick smear layer, of which the composition is a mixture of mineral and DD collagen, induced by heat/friction during cavity preparation [15]. It has been reported that, after acid etching, the organic debris in the smear layer and the smear plugs in tubules could be largely removed or rinsed off; however, using in situ micro-Raman mapping technique, Wang and Spencer have reported that the disorganized/denatured collagen in contact with the dentin substrate could not be removed [16,17]. Together with trapped residual mineral, the denatured, gelatinized collagen could inhibit the formation of an impervious seal at the dentin–resin interface [16,17]. The existence of structurally altered DD collagen has also been frequently reported in caries-affected dentin substrates and non-carious sclerotic lesions [18,19,20,21], which dentists often deal with in dental practice. These clinically relevant substrates have distinct physical, chemical, and mechanical changes different from sound/natural dentin making them challenging substrates to work with [21,22,23]. Within these clinical substrates, collagen denaturation status is very often unknown or undetermined, and the changes are usually not uniform or are strongly dependent on carious or other disease processes or the history of the substrates. In addition, current techniques, such as transmission electron microscopy or differential scanning calorimetry, have been used to characterize collagen denaturation and are often sophisticated, requiring special equipment or specimen preparation and/or homogeneity. Thus, there is a lack of simple and quantitative characterization methods that allow the in situ assessment of dentin collagen denaturation status. Due to these challenges, there are still many unanswered questions, such as the unclear relationship between collagen denaturation status and exogenous crosslinking treatment effects or the ability of different crosslinkers to enhance/restore the properties of DD collagen.

In this study, two experimental methods (heat and acid denaturation) were used to induce DD collagen in order to achieve a homogenous and characterizable substrate, and to carry out controlled quantitative evaluations of the effect of different crosslinkers. A fluorescein-conjugated collagen hybridizing peptide (CHP) staining technique was used with a regular light microscope to provide a simple, quantifiable method for collagen denaturation. CHP is a new and inimitable synthetic peptide that can particularly bind to unfolded or denatured collagen strands/chains via hydrogen bonding [24]. The objectives of this study were to assess the denaturation status of dentin collagen using various techniques, including CHP, and to evaluate the effects of three crosslinkers (EDC/NHS, CR and TF) on the structure, biostability against enzymatic degradation, and mechanical properties of natural and DD collagen. The null hypotheses of this study were (1) that DD collagen would have similar structural characteristics and CHP fluorescence intensity to natural dentin collagen, and (2) that regardless of the type of crosslinkers used, crosslinker treatment would not improve biostability against enzymatic degradation or the mechanical properties of natural and DD collagen.

## 2. Materials and Methods

All reagents used in this study were purchased from Sigma-Aldrich (St. Louis, MO, USA) unless otherwise noted. Crosslinker TF from black tea extract was purchased from Lisi (Xian) Bio-Tech Inc. (Baoji City, China) and CR from Ocean Spray (Lakeville-Middleboro, MA, USA). A solution of 0.96% phosphate buffered saline (PBS, pH = 7.4) was prepared using Dulbecco’s PBS packet (P3813) and 0.002% sodium azide was added to prevent bacteria or fungi growth. TESCA buffer was prepared by dissolving 5.75 g of TES (Lot 103181, Fisher Scientific, Pittsburgh, PA, USA), and 26.5 mg of CaCl_2_ (Lot 876772, Fisher Scientific) in 500 mL of distilled water, and the pH was adjusted to 7.4 using NaOH. For collagen digestion, bacterial collagenase (from clostridium histolyticum—type I, ≥125 CDU/mg solid) was dissolved in TESCA solution. The collagen hybridizing peptide (CHP) was purchased from 3Helix (Salt Lake City, UT, USA).

### 2.1. Preparation of Dentin Collagen Specimens: Demineralization and Denaturation

**Figure 1:**
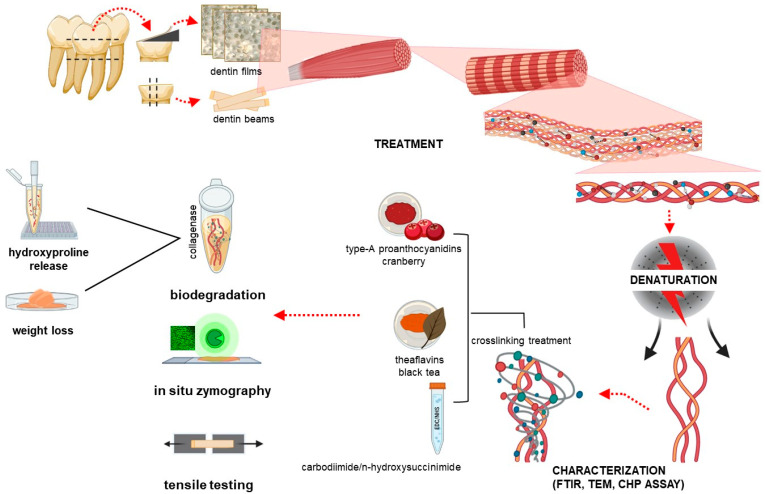
Schematic illustration of the experimental design and characterization flow chart. FTIR: Fourier transformed infrared spectroscopy; TEM: transmission electron microscopy; CHP: collagen hybridization peptide.

Fifteen non-carious human third molars were collected without any associated patient identifiers according to a protocol approved by the University of Missouri–Kansas City Adult Health Science Institutional Review Board (IRB# 12-50-NHSR). These teeth were cleaned of any remaining soft tissue and were stored in PBS containing 0.002% sodium azide at 4 °C. Roots, enamel crown and surrounding enamel of the teeth were removed using a water-cooled low speed diamond saw (Buehler, Lake Bluff, IL, USA) to achieve dentin blocks measuring 7 mm × 7 mm × 2 mm. The dentin blocks were further cut into two different types of samples: dentin beams with dimensions 7 mm × 0.8 mm × 0.8 mm and dentin films with dimensions 7 mm × 7 mm × 7 µm. Dentin films were cut using a tungsten carbide knife mounted on microtome (SM2500S, Leica, Deerfield, IL, USA). The dentin films were demineralized with 10% phosphoric acid for 30 min, while the dentin beams were demineralized for 5 h and the solution was changed 3 times. All specimens were then rinsed with deionized (DI) water 3 times for 10 min each. Complete demineralization was verified by FTIR. The dentin collagen specimens were randomly divided into 3 groups (natural, heat denatured, and acid denatured). Natural dentin specimens were immersed in 0.96% PBS. For heat denaturation (HD), the demineralized dentin specimens were heated in a water bath at 100 °C for 1 h. For acid denaturation (AD), the demineralized dentin specimens were placed in 35% phosphoric acid for 3 h at 37 °C. Each specimen was then rinsed with DI water 3 times for 30 min. The experimental design and characterization flow chart is shown in Figure 1.

### 2.2. Collagen Crosslinking Treatment

Crosslinkers TF, CR, EDC/NHS (in a ratio of 0.05 M:0.02 M) were tested and untreated dentin collagen specimens were used as control (CT). All crosslinkers were dissolved in DI water to achieve a final concentration of 1%. Dentin collagen films from the 3 main groups (natural, HD and AD) were rolled on to a cover slip with the help of a small brush and each film was immersed in its respective crosslinker solution for 30 s or 1 h. The films were then rinsed with DI water 3 times for 30 min. Dentin collagen beams were immersed in the crosslinker solutions for 1 h, followed by rinsing with DI water 3 times for 30 min.

### 2.3. Transmission Electron Microscopy (TEM) Analysis

Collagen structure with details of the bending structure of collagen was analyzed to characterize DD collagen specimens. Dentin collagen beams (n = 2 per group) from the 3 types of collagen specimens (natural, HD and AD) were fixed in 2.5% glutaraldehyde buffered with 0.1 M sodium cacodylate for 1 h, and dehydrated in graded solutions of ethanol (33%, 67%, 85%, 95%, 100%) for 2 h followed by post-fixation with 1% OsO_4_ for 1.5 h and dehydrated in graded solutions of ethanol (33%, 67%, 85%, 95%, 100%) for 2 h each. The specimens were then treated with 1:1 solution of ethanol and propylene oxide for 30 min, followed with 100% propylene oxide for 2 h, and finally with 1:1 solution of propylene oxide and epoxy resin (Embed-812, Electron Microscopy Sciences) overnight. After final infiltration with pure epoxy resin, specimens were incubated in an oven at 60 °C for 48 h. Ultrathin (60–100 nm) sections were cut with an ultramicrotome (Leica, Buffalo Grove, IL, USA). Each section was stained with 2% uranyl acetate and observed under TEM (FEI, Hillsboro, OR, USA) at an accelerating voltage of 80 kV.

### 2.4. Fourier Transform Infrared Spectroscopy (FTIR) Analysis

Chemical and secondary structural changes associated with collagen denaturation and biomodification were detected with FTIR. The crosslinker-treated and untreated dentin collagen films (n = 5 per treatment group) from the 3 main groups were spread on a cover slip and were allowed to dry in vacuum for 24 h. The FTIR spectra of the films were collected at a resolution of 4 cm^−1^ with 64 scans using FTIR spectrometer in transmission mode on a barium fluoride disk using the Perkin Elmer FTIR Spectrum Spotlight system (Spectrum one, Spotlight 300, Perkin Elmer, Waltham, MA, USA). The attenuated total reflection (ATR) mode was used to get the spectra of the crosslinker powders used. The spectra were analyzed using Spectrum Software (version 5.3, Perkin Elmer). Two-point baseline correction and normalization to amide I band was undertaken, and band ratios were calculated. For DD CTs the band height ratios A1235/A1445 were measured, while the band ratios (A1400/A1450 and A1550/1450) of spectra of all specimens were calculated. The ratios of band area of the two crosslinkers (~1145 for TF, ~1120 for CR) and Amide I band height were also quantified to evaluate the extent of interactions with dentin collagen. Amide I band height was chosen as it was not affected by heat or acid denaturation.

### 2.5. Collagen Hybridizing Peptide (CHP) Assay

Characterization of natural and DD collagen was undertaken using a CHP assay, where 60 µg CHP 5-FAM conjugate (F-CHP) purified lyophilized powder was dissolved in 400 µL pure water to make 50 µM stock CHP solution and stored at 4 °C until use. The 50 µM stock solution was further diluted to 5 µM CHP staining solution which was heated in a digital dry bath (Thermo Scientific, Waltham, MA, USA) at 80 °C for 10 min to disassociate the recombined CHP molecules in the solution, followed by quenching in ice-water bath for 10 s. Then, 20 µL CHP staining solution was quickly pipetted onto each dentin collagen film (n = 5 per group) to cover the entire surface before it was incubated overnight at 4 °C.

The stained films were then rinsed with water for 5 min three times at room temperature. After air drying, the films were mounted on a glass slide and visualized with a confocal laser scanning microscope (CLSM) (TCS SP5 II, Leica Microsystems, Buffalo Grove, IL, USA) in a fluorescence mode (20× objective lens of 0.95 NA) at an excitation of 543 nm and an emission of 549–625 nm. Three images obtained from the same z layer were randomly captured for each film. The fluorescence intensities of all images were quantified using the NIH Image J software (version 1.8.0, Bethesda, MD, USA).

### 2.6. Biostability against Exogenous Collagenase Degradation Using WL and HYP Analyses

Sixty 7 µm thick dentin collagen films were used for each treatment group. For exogenous enzymatic degradation, the treated specimens from each group were dried in vacuum and weighed using an analytical balance (Mettler Toledo AG285, Zurich, Switzerland). The specimens were then subjected to digestion by 0.1% collagenase (type I, from clostridium histolyticum, ≥125 U/mg) in TESCA buffer for 1 h at 37 °C in an incubator and the percent WL before and after digestion was calculated. Following digestion, the digestion solution was collected and hydrolyzed using 6M HCl at 110 °C for 24 h. The dry residue was oxidized and subjected to 5% Ehrlich’s reagent to develop a color. The absorbance was read at 555 nm with a microplate reader (Biotek Instruments, Winooski, VT, USA). The trans-4-hydroxy-L-proline (analytical standard) was used as the standard to create the working curves for the quantification of HYP released from each mg of dentin collagen specimens during the digestion.

### 2.7. Endogenous MMP Activity Analysis

Five dentin collagen films per treatment group were assessed for endogenous MMP activity. The collagen films were spread on a cover slip with the help of a small brush. Self-quenched fluorescein conjugated gelatin from EnzCheck™ assay kit (E-12055, Invitrogen, ThermoFisher Scientific. Waltham, MA, USA) was prepared according to the manufacturer’s instruction. An amount of 10 microliters of this fluorescent gelatin solution was spread on top of each dentin collagen specimen, incubated in a humidified chamber and stored in the dark at 37 °C for 24 h. Following incubation, the samples were rinsed with DI water on the cover slip. The amount of green fluorescence produced by the hydrolyzing effect of the conjugated gelatin by MMPs was evaluated for each specimen under a CLSM (TCS SP5 II, Leica Microsystems, Buffalo Grove, IL, USA) with an excitation wavelength of 488 nm and an emission wavelength of 530 nm. For each collagen film, three images (n = 15 images for each group) were obtained from the same z layer. Intensity of green fluorescence representing the MMP activity was analyzed and quantified using the Image J 1.8.0 software.

### 2.8. Mechanical Tensile Strength Testing

Untreated and crosslinker-treated dentin collagen beams (15 per treatment group) were used. The beams in each treatment group were stored in PBS for 24 h in an incubator at 37 °C before testing. These beams were attached to the upper and lower fixtures of the SSTM-5000 tensile tester (United Calibration Corporation, CA, USA) using cyanoacrylate adhesive (Zapit, Dental Ventures of America, Corona, CA, USA). Each specimen was subjected to a 150 lb load at a speed of 0.5 mm/min. Two tensile-related properties were obtained from the stress–strain curves. The ultimate tensile strength (UTS) of each specimen was measured by dividing the maximum force at the point of failure with the cross section area of the specimen, and the elastic modulus (EM) was determined as the slope of the linear region.

### 2.9. Statistical Analysis

The normality of distribution and homogeneity of variances were assesssed using Shapiro Wilk and Levene tests. MMP activity, WL and HYP release were analyzed by three-way analysis of variance (ANOVA) and posthoc Games–Howell test (α = 0.05). Mechanical tensile stress results were analyzed using two-way analysis of variance.

## 3. Results

### 3.1. Natural and DD Collagen Characterization

**Figure 2:**
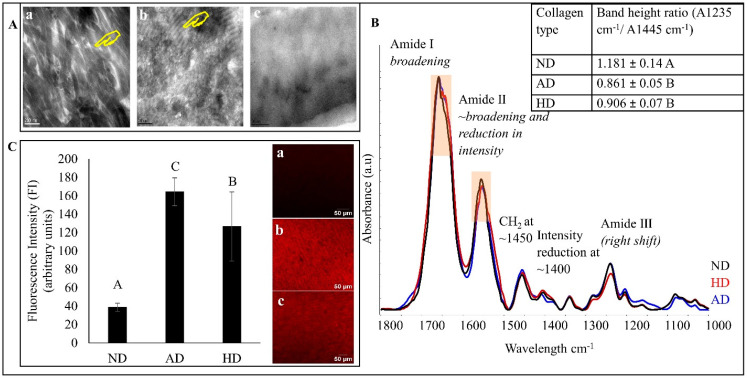
(**A**) Representative TEM images of natural dentin (ND) collagen (**a**), acid denatured (AD) collagen (**b**) and heat denatured (HD) collagen (**c**). The yellow hands show the banding pattern. (**B**) Representative FTIR spectra of all dentin collagen specimens. Natural dentin (ND) collagen in black, heat denatured in red line and acid denatured in blue line. Inset: The band height ratio (A1235/A1445) results. (**C**) Representative fluorescence images (right) and quantified fluorescence intensity (left) of all dentin collagen specimens. Means with different letters are significantly different (*p* < 0.05).

The representative TEM images of all types of dentin collagen specimens are shown in Figure 2A. Natural dentin collagen (a) shows intact fibrils with a distinct banding pattern. For AD (b), the banding pattern was not homogenous through out the specimens, while it was completely absent in HD specimens (c). Representative FTIR spectra of the three types of dentin collagen are shown in Figure 2B, where HD is represented in red, AD in blue and natural CT in black. The characteristic dentin collagen bands, including amide I (~1660 cm^−1^) associated with C = O stretching; amide II (~1555 cm^−1^) for N-H bending and C-N stretching; and amide III (~1245 cm^−1^) for C-N, C-H and N-H deformation, C-O and C-N bending at ~1400 cm^−1^ and CH_2_ scissoring at ~1450 cm^−1^, were prominent in all the spectra. A closer look revealed changes in both DD specimens, such as amide I (broadening), amide II (broadening, reduction in intensity, slight shift from ~1555 cm^−1^ to a lower wave number of ~1550 cm^−1^, decrease in intensity at ~1400 cm^−1^ δ (C-O and C-N bending)) and a reduction in intensity in amide III. The HD specimens also showed a shift to a lower wavenumber from ~1245 cm^−1^ to ~1242 cm^−1^. The inset in Figure 2B shows the band height ratios (A1455 cm^−1^/A1235 cm^−1^) of CT, HD and AD dentin collagen. Both DD types had significantly lower band ratios compared with CT (*p* < 0.001). Representative flurescence images and quantitative fluorescence intensity (FI) of CHP analysis are shown in Figure 2C where natural dentin collagen showed the lowest FI level of 38.72 ± 4.3 followed by that of HD (126.6 ± 37.5) and AD (164.43 ± 15.16), indicating a significantly higher degree of collagen denaturation by both heat and acid treatments.

### 3.2. FTIR Spectral Analysis of Dentin Collagen after Crosslinker Treatment

**Figure 3:**
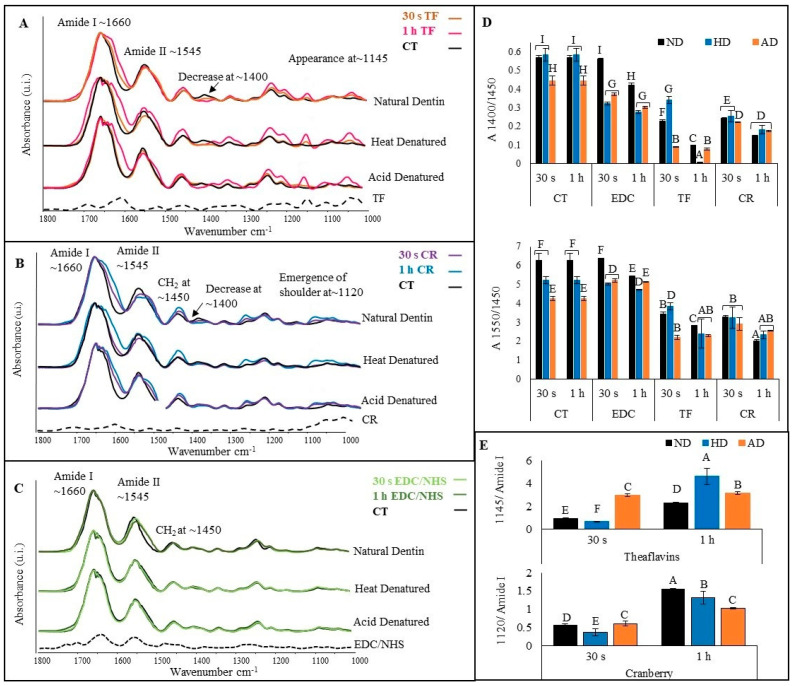
Representative FTIR spectra of TF (**A**) representing untreated control (CT) in black, 30 s in orange and 1 h in pink; CR (**B**) representing 30 s in blue and 1 h in purple; and EDC/NHS (**C**)-treated dentin collagen for 30 s in light green and 1 h in dark green. The top spectra in each box are of natural dentin collagen, the middle represent heat denatured (HD), and the bottom spectra are of acid denatured (AD). The band ratio of C-O and C-N bending to CH2 (A1400/A1450) is shown in box (**D**) at the top and that for amide II to CH2 (A1550/A1450) is shown at the bottom. The band ratio of A1145/amide I for TF-treated groups is represented in box (**E**) on top and at the bottom is the band ratio A1120/amide I for CR-treated groups. Means with different letters are significantly different (*p* < 0.05).

The representative FTIR spectra of TF (Figure 3A)-, CR (Figure 3B)- and EDC/NHS (Figure 3C)-treated natural dentin (top), HD (middle) and AD (bottom) dentin collagen are presented in Figure 3. The black dashed line at the bottom represents the respective crosslinker powder. The characteristic bands associated with dentin collagen could be assigned to all the treated specimens. A closer peek at the spectra showed pronounced changes in all the treated specimens compared with the CT, including amide I (broadening), amide II (broadening and reduction in intensity), increase at ~1450 cm^−1^, decrease at ~1400 cm^−1^ and an increase in amide III intensity. Other prominent changes included the appearance of a band at ~1145 cm^−1^ and a bulge at ~1107 cm^−1^ for TF-treated specimens (Figure 3A), corresponding to bulges seen in the TF powder at the same wave number, while the emergence of a shoulder at ~1120 cm^−1^ in CR-treated specimens (Figure 3B) corresponds to a bulge at the same wave number in the CR powder spectrum (represented by *). All of these changes were more intense following 1 h TF or CR treatment. On the other hand, the spectral changes of EDC/NHS-treated dentin collagen (Figure 3C) were negligible except in natural dentin following 1 h exposure.

To further characterize the collagen crosslinking interactions, the band ratios at A1400/A1450 (Figure 3D (top)) and A1550/A1450 (Figure 3D (bottom)) were measured. Irrespective of the dentin collagen type and treatment time, TF- and CR-treated dentin collagen showed a statistically significant reduction in both ratios (*p* < 0.001), indicating higher crosslinker interactions with dentin collagen. To quantify the extent of TF and CR interactions with dentin collagen, the band ratios at A1145/amide I for TF (Figure 3E (top)) and A1120/amide I for CR (Figure 3E (bottom)) were measured. The ratios of TF- and CR-treated dentin collagen were significantly higher (*p* < 0.001) when treatment time was increased from 30 s to 1 h, irrespective of dentin collagen type.

### 3.3. Biostability Using WL and HYP Analyses

**Figure 4:**
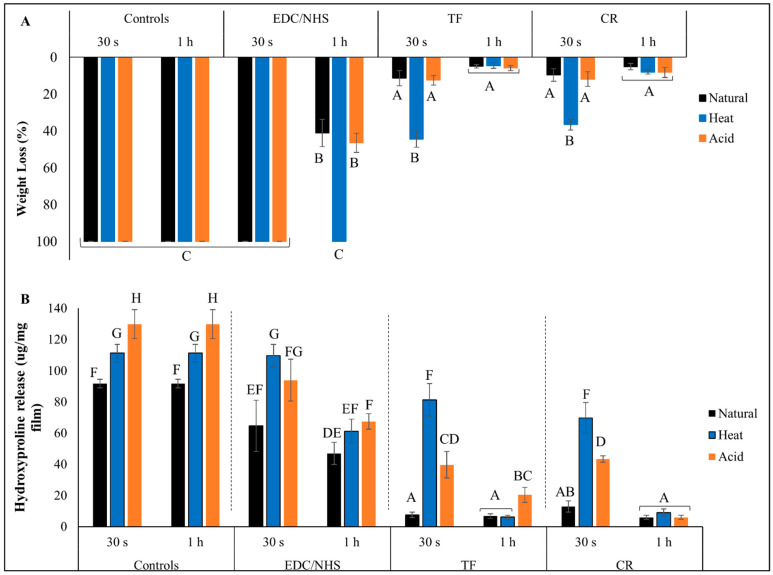
Percent weight loss (**A**) and hydroxyproline release (**B**) of controls and crosslinker-treated groups after collagenase digestion. Means with different letters represent significant differences within and between experimental groups (*p* < 0.05).

Results for collagen biostability against exogenous collagenase degradation are presented in Figure 4. Lowest WL (~10%) was seen in TF- and CR-treated groups irrespective of treatment time or collagen type, indicating the highest collagen biostability. This was true with the exception of the 30 s treated HD groups (~35% WL), where a reduction in biostability was observed (*p* < 0.0001). For EDC/NHS, only the 1 h treatment of natural and AD dentin collagen showed some biostability (~45% WL). Highest WL (100%) and complete collagen digestion was observed for all other EDC/NHS groups, which was comparable to all CTs (*p* > 0.05). Similarly, lowest HYP release (~5–~20 µg/mg) was seen in TF- and CR-treated groups irrespective of treatment time or collagen type (Figure 4B), except for the 30 s treated denatured collagen groups, where there were higher HYP releases (~75 µg/mg for HD and ~40 µg/mg for AD, respectively) observed (*p* < 0.0001). For EDC/NHS, only the 1 h treatment showed a reduction in HYP release as compared with respective CTs.

### 3.4. MMP Activity Analysis

**Figure 5:**
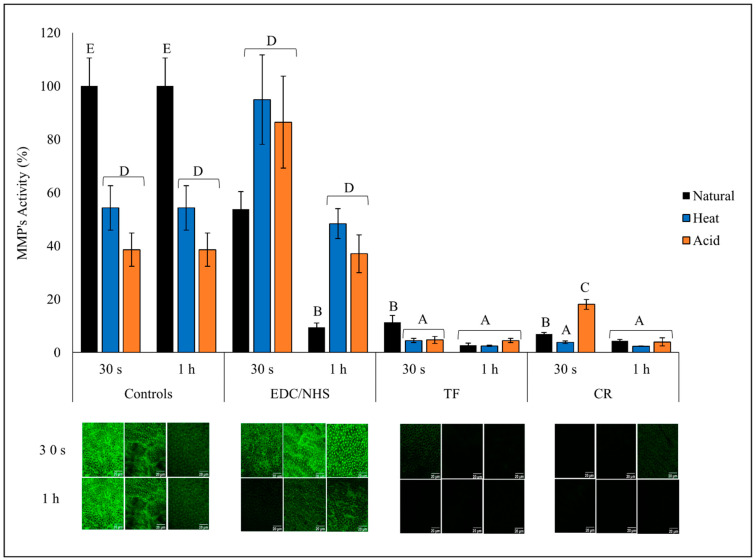
Endogenous MMP activity of all treatment groups. Relative green fluorescence intensity representing MMP activity (top) and representative images (bottom) of controls and treated dentin collagen following 24 h incubation in quenched fluorescein-labeled-gelatin. The images were obtained with a CLSM using green channel showing fluorescence attributed to MMP activity. Means with different letters are significantly different (*p* < 0.05).

The green fluorescence intensity (top) representing the activity of the endogenous MMPs and the representative images (bottom) are presented in Figure 5. The untreated natural dentin collagen showed highest overall fluorescence, indicating strong MMP activity compared with AD and HD (*p* < 0.0001). Both CR- and TF-treated groups were able to reduce MMP activity by ~95% when treated for 30 s or 1 h irrespective of the dentin collagen type (*p* > 0.05), except for CR- and TF-treated natural collagen and CR-treated AD for 30 s, which reduced MMP activity by ~90% and ~70%, respectively. One hour EDC/NHS-treated natural collagen showed a dramatic reduction of ~90% compared with CT and ~70% when compared with 30 s treatment time (*p* < 0.0001); however, EDC/NHS showed almost no effect (at 1 h), or even increased (at 30 s), MMP activity in DD collagen groups.

### 3.5. Mechanical Tensile Strength Testing

**Figure 6:**
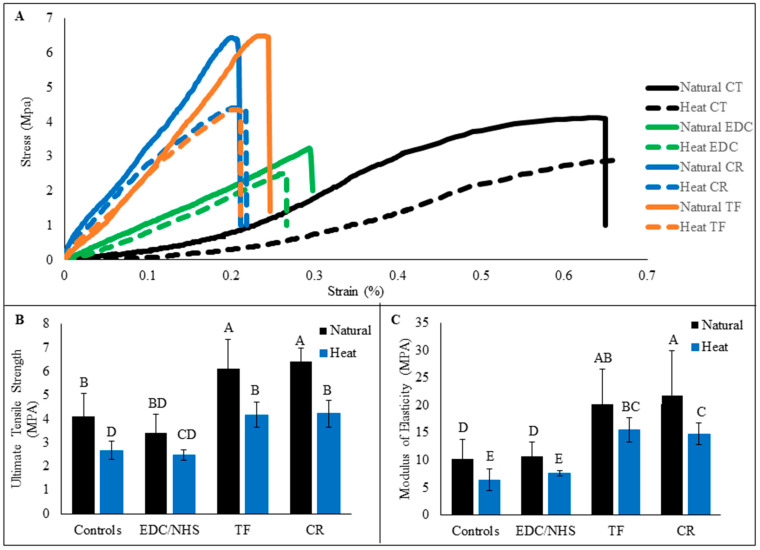
Representative stress–strain curves of natural and heat denatured dentin collagen beams from all experimental groups are represented (**A**). Collagen beams were treated with crosslinkers for 1 h. The curves of untreated controls (CT) showed typical characteristics of a soft collagenous tissue, featuring a long toe region with low and slow-rising slope. Ultimate tensile strength (**B**) and modulus of elasticity (**C**) of untreated and treated collagen beams are shown below. Means with different letters are significantly different (*p* < 0.05).

Representative stress–strain curves of dentin collagen beams before and after 1 h of treatment from all experimental groups are presented in Figure 6A. Note the obvious shape change after 1 h of treatment with all three crosslinkers, of which the slopes in the initial regions (at lower strain values) all surpassed those before treatment. The ultimate tensile strength (UTS) results are represented in Figure 6B and the elastic moduli (EM) were calculated based on the slopes in later linear regions and are represented in Figure 6C. Natural dentin collagen had significantly higher UTS and EM values compared with HD collagen (*p* < 0.05). Highest UTS and EM values were observed in TF-and CR-treated natural dentin collagen followed by TF- and CR-treated HD dentin collagen, which had UTS values significantly similar to untreated natural dentin collagen (*p* > 0.05). EDC/NHS treatment did not show any significant improvement of UTS and EM on natural or HD dentin collagen compared to control.

## 4. Discussion

Dentin has a complex chemical and physical structure made of a fibrous three-dimensional network of type I collagen that is impregnated with carbonated apatite mineral. Despite having a composition similar to bone, dentin is comparatively more interwoven, with extensive crosslinking throughout its structure [25]. The triple helical collagen molecules are stabilized through extensive hydrogen bonds as well as inter- and intramolecular crosslinks (covalent bonds) that provide stability, strength and resilience to dentin collagen [26]. However, under clinical conditions, this resilient collagen structure may be damaged or denatured due to heat, friction or mechanical forces during cutting for dental cavity preparation [15]. Dental diseases such as caries and sclerosis would also lead to disruption of collagen crosslinking, alteration of the secondary structure, and denaturation of the collagen fibers [21,27]. Reduction in pH due to either oral bacteria or during an acid etching restorative procedure leads to the further activation of dormant endogenous enzymes in the dentin matrix, which then starts a vicious cycle of collagen degradation [28].

Biomodification of dentin collagen using exogenous crosslinkers has been shown to enhance the biostability as well as the mechanical properties of sound natural dentin collagen [29]. The few studies that have evaluated the effect of the exogenous crosslinkers on clinically relevant dentin substrates have numerous limitations deriving from challenges such as unknown or undetermined collagen denaturation status or the lack of a homogenous substrate [30,31]. Hence, it is difficult to study the relationship between exogenous crosslinking treatments and collagen denaturation or to quantify their ability to enhance/restore the properties of the DD collagen. In this study, two different denaturation methods, HD and AD, have been used to produce quantifiable DD collagen substrate. To achieve the homogenous denaturation of collagen and the uniform diffusion of the crosslinkers, we used thin dentin films of ~7 µm thickness, which prevents the inhomogeneous diffusion of the crosslinker into the specimens, especially during short, clinically relevant treatment times (<1 min). In addition, the acid-etched dentin layer we tried to bio-modify through the crosslinking treatment is less than ~10 µm thick. Hence, to mimic a clinically relevant substrate, it is desirable to use dentin collagen specimens that are 7–10 µm thick.

### 4.1. Characterization of Dentin Collagen Denaturation

Characterization of the dentin collagen denaturation was undertaken using TEM, FTIR and CHP (Figure 2). TEM images indicate that denaturation causes a reduction in, or complete absence of, banding patterns and collagen fibrillar structures in DD specimens (Figure 2A). From FTIR, we can see that the most prominent changes include the broadening of amide I and a reduction in amide II intensity, which could be due to the formation of water-mediated hydrogen bonds in the DD collagen [32,33]. The shift of amides II and III to lower wave numbers can be attributed to structural rearrangements or depletion of the triple helix structure of dentin collagen following denaturation [32,34,35]. The band ratios of A1235/A1450 of AD and HD dentin collagens were 0.86 and 0.91, respectively, as compared with that of the natural dentin collagen (1.18). Dentin collagen with an intact triple helical structure has an A1235/A1450 value close to 1, while this value reduces to around 0.5 when the structure is completely destroyed and becomes similar to gelatin [36,37]. This reduction in the band ratio indicates that our denaturation techniques were able to induce structural changes/damages in the collagen, causing it to denature. The collagen denaturation status was further confirmed by a quantitative, fluorescently conjugated CHP molecular staining approach. This CHP or collagen hybridizing peptide does not bind to intact collagen chains but shows strong affinity to unfolded/damaged collagen, and thus has been used to characterize collagen denatured by enzymes, heat, or mechanical forces in various tissues [24,38,39]. Our FI values from CHP show the lowest level of 38.7 from natural collagen, a medium–high level of 126.6 from HD, followed by the highest level of 164.4 from AD, indicating that heat treatment induces a lesser collagen denaturation than acid treatment, which is consistent with FTIR results.

### 4.2. Effects of Dentin Collagen Denaturation

It would be interesting to determine how collagen denaturation affects biostability, MMP activity and mechanical properties as compared with natural collagen. While WL results do not show any difference between natural and DD collagen (*p* > 0.05), HYP release (a more sensitive molecular technique) was significantly higher in DD compared with natural dentin collagen (*p* < 0.001). Structurally altered and unwound triple helix structure of DD collagen provide more cleavage sites for collagenase to act on, allowing them to be broken into smaller peptide units, causing a higher HYP release [40]. A significant difference in the FI (Figure 2C) of natural collagen and of both DD specimens (*p* < 0.0001) also shows that denaturation caused an unwinding of the triple helix structure, and hence provided more sites for the fluorescently conjugated CHP to bind to [38,39]. Based on these results, the first hypothesis is completely rejected. Both denaturation techniques used in this study alter the collagen structure in their own unique way. Heat causes collagen structural alterations which are accompanied by gross shrinkage of the whole structure [41], while acid triggers hydrolysis of the collagen molecule, causing it to breakdown into smaller peptides that provide more sites for CHP molecules to bind to and that also cause a gross loss of collagen substrate [42]. This is why we see the highest HYP release, as well as the highest FI, in AD dentin collagen.

Endogenous MMP activity was reduced in both DD samples compared with natural dentin collagen (*p* < 0.001). This can be explained by the probable denaturation of the MMP enzymes themselves, hence affecting their ability to cleave collagen [43]. Another explanation could be the alterations of the specific cleavage sites on the collagen molecule itself, such as Gly-Ile in α1 chain and Gly-leu in α2 [44] upon which the enzymes act. These alterations limit the ability of MMP to bind to and cleave the collagen molecule.

The triple helix structure in natural dentin collagen is packed into a tight coil with extensive crosslinks, making it a resilient structure [45]. Denaturation would cause loss of crosslinks and unwinding of the triple helices which would affect the mechanical integrity of dentin collagen [5,46]. A significant difference was observed between the mechanical properties of natural dentin and HD collagen (*p* < 0.001) in this study. This further supports our HYP release (Figure 4B) and DD collagen characterization (Figure 2) results, implying that structural alterations and unwinding of the triple helix structure of collagen reduces the mechanical properties of dentin collagen following denaturation. As explained earlier, the nature of the AD process causes hydrolysis of collagen, which leads to loss of the dentin collagen substrate. Hence only HD dentin collagen beams were used to compare mechanical properties.

### 4.3. Effects of Different Crosslinkers

The effects of different crosslinkers on the molecular structural changes of dentin collagen were evaluated by FTIR, which showed distinct spectral changes in natural and DD collagen before and after treatment with the different crosslinkers. Evidence of hydrogen bonding (between amino and amide groups of collagen and phenolic (OH) groups of TF and CR) is seen in all dentin collagen substrates treated with TF and CR, represented through spectral changes in amide I (broadening) and amide II (broadening and reduction in intensity) [47]. Another predominant change was the reduction of the ~1400 cm^−1^ band intensity due to hydrophobic interactions causing dehydration via the replacement of bound water in collagen by TF and/or CR [48,49]. The spectral changes induced by the crosslinkers were quantitively measured with A1400/A1450 and A1550/A1450 ratios (Figure 3D). Reduction in the ratio is attributed to high collagen/crosslinker interactions. The crosslinkers of the TF- and CR-treated groups showed the lowest ratios compared with the controls. Increased interactions translate into the induction of more crosslinks and improved protections against enzymatic degradation, which can be confirmed through the reduced WL and HYP release (Figure 4) values of the TF- and CR-treated groups. To further characterize the extent of collagen interactions of TF and CR, ratios of the specific contributory bands of the two crosslinkers and amide I were measured (Figure 3E). An increase in the ratios means higher collagen/crosslinker interactions. Irrespective of the dentin collagen type, both ratios significantly increased following 1 h treatment.

The changes in the FTIR spectra of the EDC/NHS-treated dentin collagen (Figure 3C) were less intense as compared with TF and CR, which included a reduction of the amide II band, which could be due to a decrease in -NH_2_ in response to increased crosslinking [50,51]. Unlike TF and CR, which can induce crosslinking through various mechanisms, EDC/NHS is a zero-length crosslinker that acts through the activation of the carboxylic acid groups in collagen, which in turn form an intermediate product (O-acylisourea) that slowly reacts with amino groups in collagen to form amide bonds [52]. Hence, EDC/NHS acts by facilitating the formation of amide bonds without getting incorporated into the collagen, as is evident in the FTIR spectra which show the negligible contribution of EDC/NHS powder to the treated collagen. The band ratios (Figure 3D top) for EDC/NHS showed a significant reduction (*p* < 0.001) following 1 h treatment time compared with the controls. This is due to a reduction of band intensities at ~1400 cm^−1^ and amide II (Figure 3C), caused by the formation of amide bonds between carboxylic acid and amines groups within the collagen molecules.

The effects of different crosslinkers on the resistance to exogenous enzymatic degradation of DD collagens were also assessed. Effective crosslinking should strengthen the triple helix and prevent unwinding of the collagen fibrils by sterically blocking the cleavage sites to bacterial collagenase or by directly inhibiting these enzymes. For natural collagen, TF or CR treatment always resulted in a significant reduction of WL and HYP values compared with untreated natural collagen (irrespective of treatment time). However, for DD collagens, TF or CR caused a lesser reduction in WL and HYP values after 30 s treatment, although the values were still significantly reduced as compared with untreated controls. DD collagens showed dramatically increased resistance only after 1 h treatment. It is likely that increasing the treatment time to 1 h gives the crosslinker more time to diffuse through the collagen structure and induce additional crosslinks to the unwound DD collagen, hence providing it higher resistance against collagenase degradation. This is in agreement with the A1145/amide I and A1120/amide I ratios (Figure 3E) which indicate higher collagen/crosslinker interaction after 1 h treatment time.

Compared with TF or CR, EDC/NHS treatment provided little or much less protection to DD or natural collagen against collagenase digestion under similar conditions. For example, the results show no protection against collagenase after 30 s, and only the 1 h treatment time showed a reduction in the WL and HYP values of all dentin collagen types (except WL of HD) as compared with untreated controls. EDC/NHS activation results in the formation of intra- and interhelical crosslinks instead of intermicrofibrillar, which can be detected by sensitive techniques such as HYP analysis, but not by WL [53]. This would explain why we could see significantly higher WL for EDC/NHS-treated HD but similar HYP values for both DD. Being a zero-length chemical crosslinker, the mechanism through which EDC/NHS works is slow and would produce better results following longer treatment time [54]. However, some studies have also shown that the crosslinking activity of EDC/NHS reaches a plateau after 1–2 h treatment time, which could be due to the limited availability of carboxyl groups on collagen for interaction or limitation of EDC/NHS to access the reaction sites [12]. Unlike TF and CR, which have more diverse mechanisms by which to interact with collagen, the limited crosslinking potential of EDC/NHS results in comparatively poor protection against collagenase digestion.

Both TF and CR were able to reduce the MMP activity of DD collagen irrespective of the treatment time and in fact showed significantly higher reduction in MMP activity than crosslinker treated natural collagen. Hence, beyond the introduction of extensive crosslinks which could sterically block the active sites on MMPs, both TF and CR can also directly inhibit MMPs and protect dentin collagen from degradation [9,55]. Ability of EDC/NHS to form inter- and intrahelical bonds allows it to crosslink dentin collagen as well as the dentin-matrix-bound MMPs [56]. The carboxyl and amino groups in MMPs are more accessible than those in collagen, making EDC/NHS a potent MMP inhibitor than a collagen crosslinker [57]. This explains the dramatic reduction of MMP activity in natural dentin collagen following 1 h EDC/NHS treatment. Surprisingly, our studies have shown for the first time that the MMP activity of EDC/NHS-treated DD collagen was even higher (30 s) or the same as (1 h) that of untreated controls, indicating that EDC/NHS was not able to inhibit MMP activity as it was for natural dentin collagen. Following denaturation, alterations of the triple helix structure as well as of MMP cleavage sites make it challenging for EDC/NHS to effectively reduce MMP activity.

Because the denaturation of dentin collagen causes a reduction in tensile mechanical properties (Figure 6), it is of vital importance to induce exogenous crosslinking which would make DD collagen stiff and provide stability against degradation. As AD might have caused an uncontrollable loss of the collagen substrate which made UTS calculation inaccurate, we used HD dentin collagen beams only in tensile testing. Regarding tensile properties, three crosslinkers had a positive effect, showing the initial slopes at the toe region or low strain values exceeded the ones before crosslinking treatment. There was significant improvement in the tensile properties of both TF- and CR-treated natural and HD dentin collagen compared with untreated controls. However, EDC/NHS-treated natural and HD collagen did not show much improvement in UTS and EM but did show a reduction in tensile strain as compared with controls. The difference in improvement of tensile properties may be due to the more diverse crosslinking interactions available in TF/CR vs limited crosslinking activity in EDC/NHS, as explained above. Hence, the second hypothesis was also rejected as TF and CR proved to be promising crosslinkers, able to improve the biostability and the mechanical properties of natural and denatured dentin collagen.

The limitations of this study may include that it is unclear if denatured collagen produced by the two experimental protocols would be comparable to that caused by clinical procedures or carious activity. The focus of forthcoming studies would be on the study of the direct effects of these crosslinkers on collagen in clinically relevant substrates, including caries-affected and sclerotic dentin. The CHP molecular staining technique will be used for the in situ monitoring of collagen alteration status in these substrates. The MMP activity and biostability of the same substrate sections will be characterized. For example, it would be interesting to verify the loss in the MMP inhibition function of EDC/NHS on altered collagen in these clinically relevant substrates.

## 5. Conclusions

The denaturation techniques (heat and acid) used in this study were able to induce structural changes in dentin collagen and produce a quantifiable DD collagen as verified by various techniques, including CHP staining. Dependent on the crosslinkers used, crosslinking treatment altered collagen structure and improved the biostability of denatured dentin collagen to different extents, with EDC/NHS being the least effective. Surprisingly, the well-known MMP inhibitor EDC/NHS demonstrated that it had only a minimal effect on (1 h), even prompting an increase in (30 s), MMP activity in denatured dentin collagen. It was generally more challenging to crosslink heat-denatured collagen in 30 s; however, significant improvement was seen following 1 h treatment time. Both TF and CR were effective in crosslinking denatured dentin collagen, showing even better biostability and tensile properties than natural dentin collagen when untreated.

## Data Availability

Data are available from the corresponding author upon request.

## Abbreviations

DD: denatured dentin, ND: natural dentin, HD: heat denaturation, AD: acid denaturation, CT: control, EDC/NHS: 1-ethyl-3-(3-dimethylaminopropyl) carbodiimide/n-hydroxysuccinimide, TF: theaflavins, CR: cranberry juice, PAC: proanthocyanidins, WL: weight loss, HYP: hydroxyproline release, MMPs: matrix metalloproteinases, CHP: collagen hybridizing peptide, TEM: transmission electron microscopy, FTIR: Fourier transform infrared spectroscopy, CLSM: confocal laser scanning microscope, UTS: ultimate tensile strength, EM: elastic modulus, FI: fluorescence intensity.
